# Misalignment of age clocks

**DOI:** 10.1007/s11357-025-01750-2

**Published:** 2025-06-25

**Authors:** Xiaoyue Mei, Hannaneh Kabir, Michael J. Conboy, Irina M. Conboy

**Affiliations:** Department of Bioengineering and QB3 Institute, UC Berkeley, Berkeley, CA 94720 USA

**Keywords:** Biological aging, Elastic net regression, Machine learning (ML)

## Abstract

Biological aging is a complex non-linear process, with markedly distinct starting and end points, yet the biomarkers of its progression remain elusive. A key assumption of most machine learning (ML) approaches for age clocks is that predictive biomedical features can be identified via mathematical transformations of data to favor a linear transition from start to end, even if they erase any natural biological pattern. It is given that expected correlations, e.g., time lived (age) and time left to live (mortality), would persist in such mathematically optimized models, biologically meaningful or not. Here, we further clarify the workings of the clocks, explain the trade-off between mathematical optimization and biological interpretability, and discuss a hallmark of aging, inflammaging, that age clocks struggle to detect. We expand on the negative consequences of incoherence in linear models where some DNA methylation (DNAm) features increase with aging and disease, while others correspondingly decrease, yet positive weights are assigned to both. We quantify the misalignment between major DNAm clocks and actual changes in DNAm, providing an interactive visualization of these errors for each model. We demonstrate that major conventional age clocks are both incoherent and skewed toward leukocyte fractions and that rectifying incoherence makes the model balanced and *not* skewed toward neutrophils and better detects inflammaging. We briefly outline non-linear ML age clocks and the advantages of identifying a natural trajectory of aging directly from the primary data.

**Graphical Abstract:**

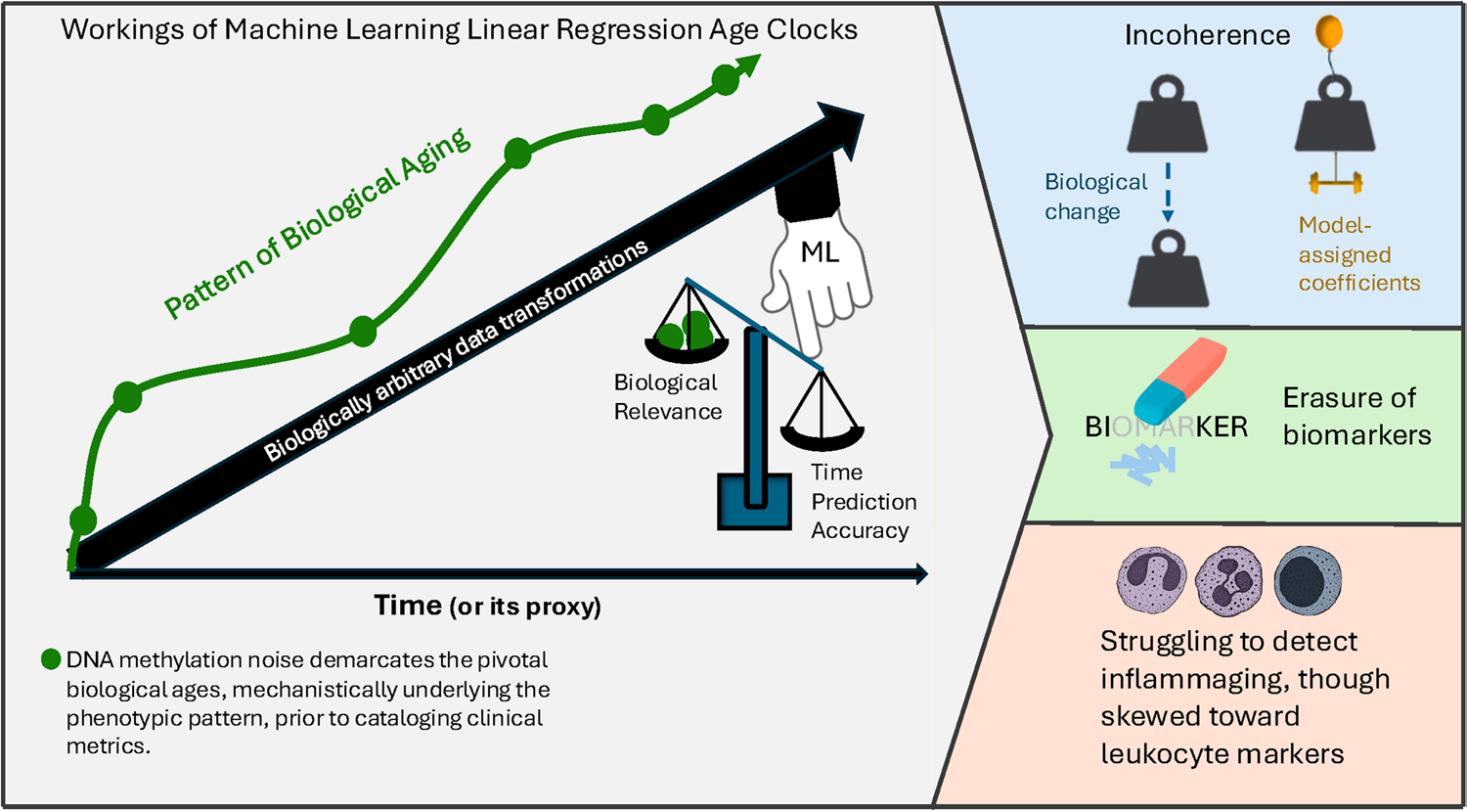

## Clocks model the transition from young to old individuals as a linear trend

**Fig. 1:**
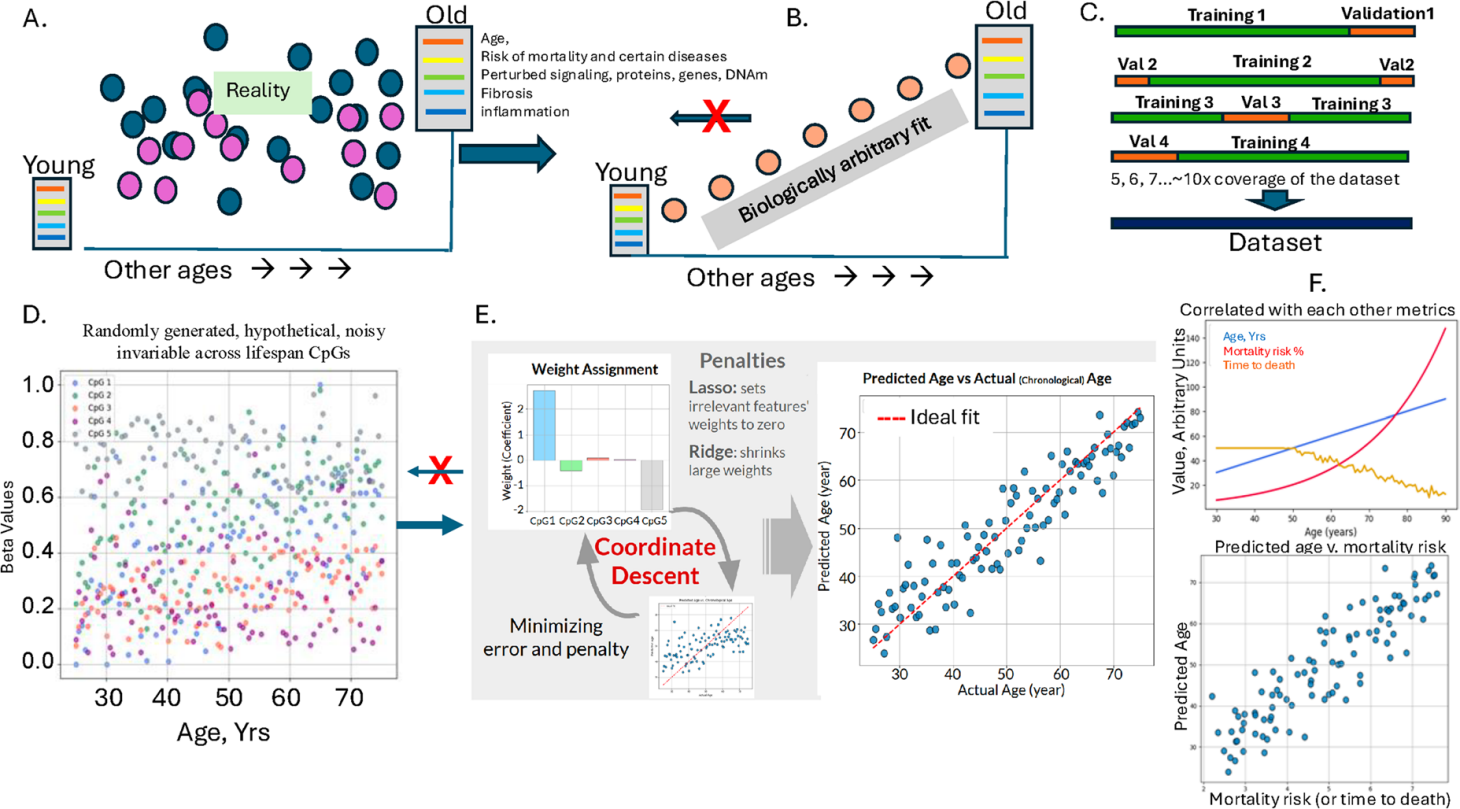
Differences between the real-life progression of biological aging and clock models. **A** Young versus old people are different from each other in many metrics, such as risk of certain diseases, perturbed signaling networks, epigenetics, transcriptomics, and proteomics, fibrosis, inflammation. Yet, the levels of most/all candidate biomarkers (circles) of biological age *progression* are highly variable and do not show a linear pattern. **B** EN clocks can be used for correlating experimental results linearly with age or its proxy, via biologically arbitrary selection, weighting and summing of a few data points; however, the process continues to be non-linear and has different numerical values in real life (crossed blue arrow). **C** Exhaustive ML rounds with ~ 5–10 × coverage of the samples’ dataset that are split to 80% for training and 20% for testing/validation. **D** Scatter plot of five hypothetical CpGs that are randomly generated to have CpG 1, 2 correlated and both equals age × 0.008 + normally distributed random noise and CpG 3 to be half of CpG 1, while CpG 4 is normally distributed and CpG 5 is uniformly distributed, and both with random normally distributed noise through chronology. **E** The steps of applying EN to the non-linear data are shown in D (blue arrow); crossed blue arrow indicates that this is one-directional transformation. **F** Illustration of de-facto correlations between metrics that already correlate with each other

Chronological age refers to the number of years that one has lived, whereas biological age reflects the degree of tissue, cell, and molecular damage, or health decline, during those years. With respect to biological age, hundreds of known biomarkers differentiate older individuals from younger ones at the functional, histological, cellular, and molecular levels. These include physical fitness, cognitive capacity, levels of CRP (a marker of inflammation), levels of cholesterol, tissue fibrosis, mitochondrial health, and many others. In contrast, after decades of collective search, including GWAS and EWAS, the consensus is that genetic and environmental variations from person to person are greater than the gradual non-linear *progression* of biological aging (Fig. 1A).

Ideally, specific biomarkers—such as the DNA methylation status at given loci, chromatin state, mRNA or protein levels, and pathway activity—would be identified as exhibiting a consistent linear change from youth to middle age to old age. These biomarkers could then serve as reliable indicators for monitoring biological age and evaluating longevity therapeutics. However, while chronological age progresses strictly linearly, biological aging is non-linear [1, 2], and our attempts to reconcile the two have not met with success.

Linear regression-based age clocks can approximate linear progression from young to old by applying mathematical transformations that filter and weight experimental values, in a biologically arbitrary way (Fig. 1B). The most commonly used method for constructing these clocks is elastic net regression (EN), a penalized linear regression model developed in 2005 by Zou and Hastie [3]. Age clocks using such models claim to delineate the risks of health decline, the effectiveness of biomedical interventions, and also to reveal the biological mechanisms that drive accelerated aging, all via quantifying the deviations from clocks’ predictions. These claims, however, are contradictory to clocks’ methodology and to their performance in fail-testing [1, 2].

EN models are typically applied to large data from DNAm hybridization arrays, in which the strength of hybridization is detected via fluorescence intensity, and the signal of each cytosine-poly-guanine (CpG) probe is divided by the sum of the methylated and unmethylated signals. This yields beta values, ranging from 0 to 1, that correct for the experimental differences in batches of reagents, instrument performance, etc., because the overall fluorescence, which can be greater or lesser in different experiments, is the denominator for each probe.

Beta values also allow comparing the results from different studies on the same relative scale, where beta values between 0 (unmethylated) and 1 (methylated) represent a combination of heterozygosity, proportions of cells, signaling dynamics, and imprecisions in hybridization. Hence, a beta value is a probability that a CpG is methylated and attempts to “normalize” the bulk data for cell heterogeneity [4] might alter several patterns. Model building, testing and validating performance, and parameter optimization involve partitioning the data into equal subsets (folds) and performing different 80/20-fold training–testing splits 5–10 or more times, to overlap the entire dataset (Fig. 1C).

EN models typically select a sparse subset of several hundred CpG sites, out of ~ 500,000 – 800,000 array probes, by shrinking coefficients to zero for the rest of the CpG sites. The goal is to assign weights to these selected sites so that the weighted sum of their beta values equals 20 for a 20-year-old, or a health score of 20, 31 for a 31-year-old, or a health score of 31, etc. However, the actual DNAm values do not exhibit linear changes, and linear regression models, such as elastic net regression, do not imply causation in natural processes [5]. Figure 1D, E illustrate how processing of DNAm data via EN model can obscure the underlying biological patterns.

A greater correlation coefficient (r) or low mean absolute error (MAE) does not signify the biological relevance of a model, because they are mathematical evaluations of model fit *after* the above *biologically arbitrary* data transformations. Biological relevance would be demonstrated if the model residuals, i.e., predictions of age acceleration, or consistent linear shifts at 2–4-year intervals, corresponded to statistically significant changes in the mRNA levels of the clock genes and/or other epigenetic changes at these CpG loci, such as histone modifications. These empirical verifications are lacking.

On the other hand, as expected, all metrics that correlate with each other that differ significantly between the younger and older individuals remain de-facto correlated, e.g., age, risk of mortality and morbidity, cell signaling intensity, levels of proteins, DNAm, or clinical parameters, e.g., CRP, cholesterol, or environmental factors (Fig. 1F). Indeed, the general reproducibility of clock approximations and the constant emergence of new clocks are based on the fact that there are many biomedical metrics which are consistently and non-randomly different between the first and the last points: young and old.

## To minimize errors in predicting time progression, EN clocks actually exclude biomarkers that reflect the underlying biology of aging

A deeper analysis of EN clocks revealed that CpG biomarkers of *biological* age are more likely to be excluded rather than selected by the L1 feature selection mechanism of elastic net regression [1]. Understanding this requires clarifying why differentially methylated cytosines—the key biomarkers for distinguishing healthy and pathological aging from DNA methylation array data—may not be effectively captured by the elastic net cost function. In short, the clock training process removes signs of individual pathologies.

**Fig. 2:**
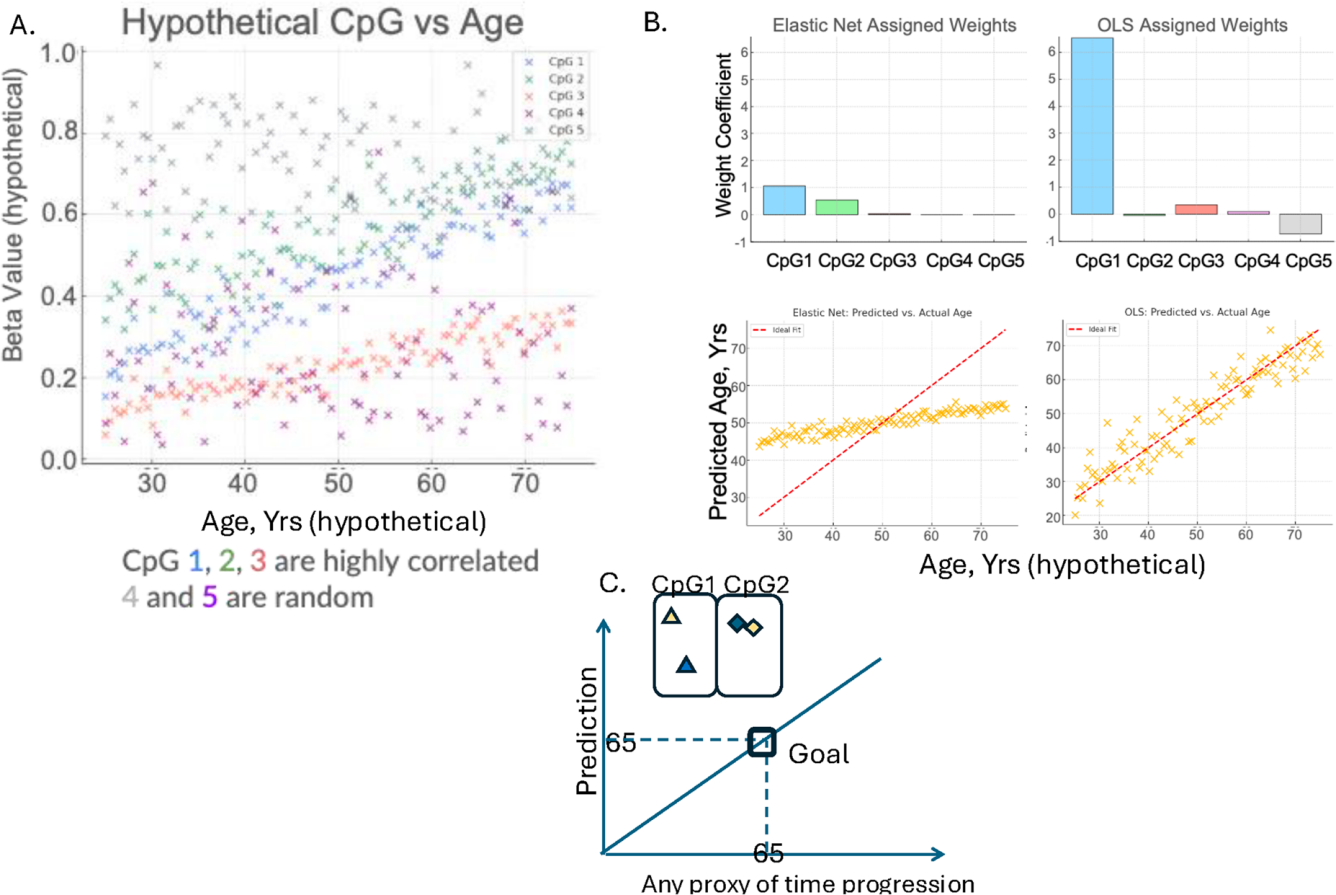
Inherent errors of the clocks. **A** Illustration of five hypothetical CpG methylation beta value patterns, where CpGs 1, 2, and 3 are collinear and correlate with chronological age, while CpG 4 is randomly distributed and CpG 5 follows a uniform distribution, both 4 and 5 exhibiting no correlation with age. **B** Application of EN and OLS to the randomly generated CpGs and ages. EN combines L1 and L2 regularization to shrink and select features, resulting in sparse coefficients, particularly in the presence of multicollinearity, while OLS fits all provided features without regularization and preserves existing collinearity. EN was under-tuned to highlight the effect of strict regularization under multicollinearity, resulting in underprediction. **C** EN clocks have the goal to fit the data from samples of a similar age (or its proxy) by finding an optimal line, generally speaking, that minimizes residual error and shrinks feature weights through regularization. Accordingly, from the two CpGs (different, triangle v. similar, diamond) between the samples of the same age (yellow and blue), the EN model tends to assign smaller or even zero weights (w) to the CpGs that diverge in the samples of the same age or its proxy, even though they can potentially be biomarkers of biological age

Most DNAm clocks are trained using elastic net regression to predict time progression, such as chronological age, or a related proxy. However, if the goal is to identify CpG biomarkers that track biological aging, elastic net may not be the appropriate model. The optimization problem it solves is structured to minimize squared residuals while simultaneously shrinking weights to prevent overfitting. The illustration in Fig. 2A, B is intended to demonstrate how EN shrinks the weights of collinear features as compared to ordinary least squares regression, OLS. However, if we change alpha to 0.001, the penalty will be very small and EN will behave like OLS. L1/L2: lasso/ridge ratio can be set to 0.8/0.2, also making the model similar to OLS. Of note, these adjustments would effectively negate the need for EN complexity, reducing its operations to those akin of OLS.

Overall, minimization of residuals introduces a fundamental incompatibility with the goal of selecting differentially methylated cytosines that distinguish healthy from pathological aging. Methyl cytosines indicative of biological aging tend to exhibit high variance across samples, in relatively healthy individuals and, particularly, those who are developing or have already developed a disease [6]. In an elastic net framework, this variability disrupts the minimization of residuals, as these CpGs may not contribute consistently to reducing error across the training set.

Worse, when a subgroup in the training data exhibits residuals that effectively cancel out when aggregated across all samples, the total residual associated with a given CpG may appear negligible. Under coordinate descent, this results in the corresponding weight being driven to zero, effectively excluding that CpG from the model (Fig. 2C). Thus, while elastic net successfully identifies CpGs that predict chronological age and all its correlations with low variance, it systematically eliminates CpGs whose biological relevance is tied to their variability—precisely those markers needed to distinguish the perturbations in healthy aging.

As the result, residuals—the deviations from the linear model in DNAm clocks—are most likely to reflect a host of non-biological factors, such as inaccuracy, overfitting, or experimental errors. These deviations, when under ± 10 years, are within the range of normal biological variation or random noise [1]. It is likely that alternative approaches, incorporating methods less constrained by residual minimization, are necessary for detecting CpGs linked to biological aging.

## Clocks struggle to detect the robust hallmark of aging-inflammaging, which should serve as an internal control for the peripheral blood datasets

Determinations of human biological age typically rely on datasets, which were generated from blood cells, e.g., peripheral mono-nuclear cells (PBMCs) or leukocytes. It is well known that gene expression in individual cells as well as the composition of blood cells changes with aging and even more so with age-related inflammatory diseases, and this is reflected in the DNA methylation [7, 8]. In the 1990s, it was found that with aging the long-term HSCs decline while the relative numbers of HSCs increase that are short-term, rapidly proliferating, and prone to myeloid differentiation [9]. In mid-2000s, an elegant study with the Sleeping Beauty transposon discovered that it is not the stem but progenitor cells that largely maintain blood lineages under physiologic conditions [10]. Yet, consistently with the previous conclusions, the propensity for myeloid differentiation was shown to increase from aging-exacerbated inflammation [11].

This lymphoid to myeloid shift, as well as the changes in gene expression reflecting the function of individual leukocytes, defines the robust hallmark of aging known as inflammaging [12]. Inflammaging prematurely ages multiple tissues and exacerbates numerous pathologies that increase in later years of life, including the risk of infections and flares of cancers, tissue degenerative diseases [13], and autoimmune diseases [14].

An easy task for a biologically relevant DNAm predictor that relies on blood cells would be to detect inflammaging, because it is reflected both in the changes of DNAm of the same cell-type and in the shifts in cell types. However, when systematically tested, all examined clock models either failed or struggled to distinguish the clear hallmark of inflammaging from healthy aging [1, 2]. Namely, predictions of age overlapped for healthy people and patients with different *chronic* inflammatory diseases, which increases with aging and which prematurely age tissues, e.g., rheumatoid arthritis, multiple sclerosis, Parkinson’s disease, or tauopathies [15–18].

Even the PACE clock that is trained on the disease scores of people of the same age struggled to detect inflammaging, and yet worked somewhat as a chronological age predictor, illustrating that clocks are always de facto trained on proxies of time progression, which in PACE is being prone to strokes and heart attacks, more vs. less.

Of note, since the proportions of cell identities, and thus cell heterogeneity, *is* a feature of inflammaging and aging in general, the value of a suggestion to mathematically minimize or “normalize” against such features in a dataset used for training a model to predict biological age, is unclear [4, 19].

## There is incoherence between CpG weighting and the actual direction of biological information

In addition to the above-discussed struggles of EN clocks to identify differentially methylated cytosines, their problems with detection of inflammaging are due to the weighting of data points incoherently with respect to the direction of the shifts in DNA methylation at individual CpGs. The “incoherent” (or “misaligned”) features are defined as CpG sites that have a model coefficient sign opposite to the direction of their univariate methylation change. Such incoherence with the actual biological trend makes it harder to interpret what the clock is actually predicting. For example, suppose a model includes two differentially methylated CpGs—call them CpG_A_ and CpG_B_—playing a role in a given inflammaging pathology. Suppose that the methylation of CpG_A_ increases while that of CpG_B_ decreases in inflammaging. EN model will assign weights to both CpG beta values without regard to the magnitude and direction of the biological change information, making any biological interpretation of the residuals of such a model conceptually and factually inaccurate.

If we knew which set of genes or CpGs biomark biological aging and selected those that do not correlate inversely with each other, there might have been no problem with incoherence. But then, there would have been no need for approximated predictors. Of note, OLS does not suffer from the collinearity problem and predicts time progression, but not biological age [1].

When incoherence is rectified, linear models provide a better resolution between the residuals of healthy people versus patient populations, while simultaneously diminishing the ability to accurately predict time progression or the age of sample donors. Thus, linear mathematical accuracy declined when the model was adjusted for a better biological relevance [1]. Recognizing this trade-off is important when applying linear modeling to approximation of biological processes, i.e., the potential limitations of prioritizing statistical fit over mechanistic insights.

In a model, even a single feature with a weight that disagrees in sign with its univariate correlation versus the response variable can compromise the interpretability of the predicted value—especially if such a feature carries a large proportion of the total weight of the model. Yet, there is an apparent dismissal of these discrepancies in the field of ML clocks of all generations [4, 20]; this error has been allowed to propagate for more than a decade without corrective measures or clear interpretation.

To address this unmet need, we examined both the proportion of incoherent features in each clock and the *weights* of these misaligned features relative to the total weight in their respective models. For all the models analyzed, the percentage of misaligned features was between 23.9% and 53.8%, meaning that in some cases, over half of the selected CpG sites have coefficients with signs opposite to their univariate correlation with age. Moreover, these features accounted for 11.2% to 40.8% of the total model weight, emphasizing a high degree of biological disconnection. This prevalence indicates that incoherent features contribute to model optimization but also raises the question about the cost of including such misaligned CpGs from the perspective of biological interpretation of clock outputs. Therefore, evaluating time prediction accuracy vs. biological relevance, with and without incoherent features, is an important step before treating residuals as biological signals [1, 4].

**Fig. 3:**
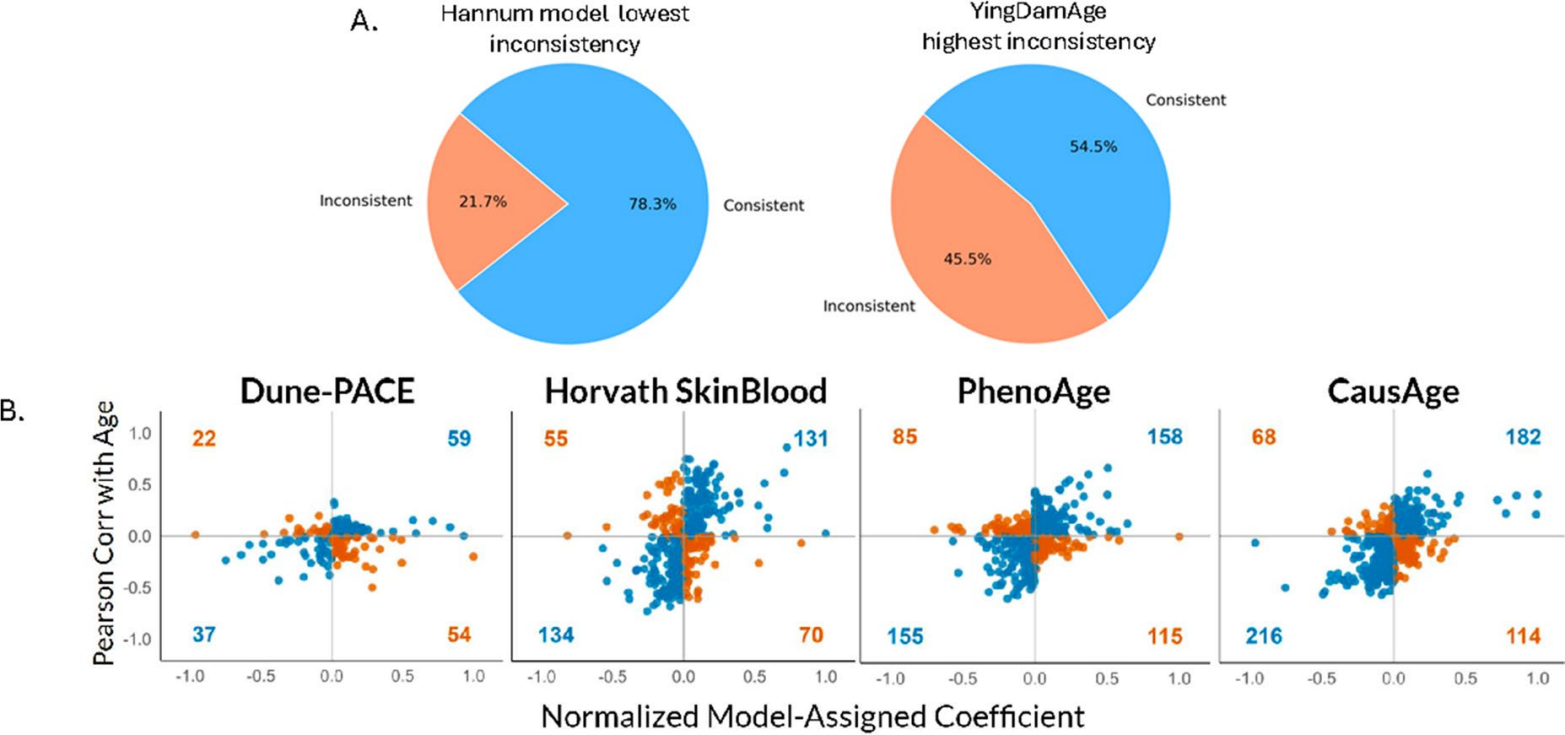
Representative images from the website that quantifies clock’s errors of incoherence. **A** Pie charts show the percent of CpGs in each model, which have their weights assignments being consistent (blue) versus inconsistent (orange) with the direction of disease-related changes in DNA methylation at these CpGs. The models with the lowest and the highest inconsistencies are shown; the rest of the models are similarly presented on the website. **B** Scatterplots display the regression model coefficients max-normalized per model (*x*-axis) against the corresponding univariate Pearson correlation coefficients for age, based on 656 individuals in Hannum et al. [21], (GSE40279) (*y*-axis). The consistency is shown through the color-coded dots: each dot signifies a CpG site that was selected by the model. CpGs with coefficients with the same directionality, both positive or both negative, are in blue, and those misaligned-inconsistent directionality are in orange

These results provide a direct look at why the first- and next-generation clocks have little to no resolution between the residuals of healthy people and patient populations. They are easily verifiable and are presented in our interactive Workings of the Clocks website. Example plots for published models from the website are shown in Fig. 3.

We have created an interactive website https://epiclockatlas.streamlit.app/ that includes all clocks up to 2024; it was generated through PubMed with the focus on whole-blood-based epigenetic clocks that have published their CpGs and other details of the models. Pie charts show the proportion of CpGs that are highly colinear and incoherent, for each epigenetic clock. The Hannum dataset was used to build univariate Pearson correlation, as it has clear labels on the health status of the sample donors [21]. Scatterplots of predicted versus actual age are shown for some models and confirm that the clocks struggle to distinguish healthy individuals from those with age-associated diseases, limiting their utility in the biomedical field. In addition, the website is also designed to serve as a comprehensive reference for researchers, as it provides summarized characteristics of each clock, including the number of features and the proxy of time progression that was the response variable. This information is also included as still images and EpiClock Excel in the Supporting Data for the Website. By consolidating these resources, the hope is to encourage the development of more biologically meaningful innovative ML approaches.

## Clocks are biased for CpGs of leukocyte subsets yet fail to detect inflammaging; a feature rectified model is more balanced and detects inflammaging better

To better understand the mechanisms by which feature rectification against incoherence of linear models enabled better resolution of inflammaging, we analyzed the abundance of the key markers of leukocyte subsets in the different clock models. As mentioned, clocks typically operate on bulk DNAm data from blood cells where the proportions of circulating cells with different fates largely contribute to the changes in beta values [1, 2, 12].

Evaluating correlation between DNAm features and markers of immune cell fractions is important, as changes in immune cell composition not only influence DNAm patterns but also reflect the health of a person. In particular, lymphocytes are needed for productive adaptive immunity, and the age-specific decline of their proportions, e.g., of naïve T-cells, is well documented and associates with many pathologies [22]. On the other hand, the numbers of myeloid cells, such as macrophages, granulocytes, and neutrophils, remain relatively stable throughout the lifespan [23, 24]. Both lymphoid and myeloid cells undergo age-specific changes in gene expression and thus in DNAm, which change even more with inflammaging [25–27]. Therefore, a biologically relevant predictor would be expected to select CpGs that have balanced DNAm signatures, which associate with the dynamics of T-cells but are not strongly linked to neutrophil or myeloid subsets.

Interestingly, suggestions were made that rectifying the incoherence of linear models causes preferential selection of CpGs that detect neutrophils, in turn detecting inflammation [4]. To resolve this point directly, we analyzed the relative abundance of CpGs that correspond to different subsets of leukocytes in the conventional clocks and in the feature-rectified model (IR-mFSS) [1].

The correlation between IR-mFSS-selected CpGs and immune cell fractions was quantified by first computing baseline variance inflation factor (VIF) without cell fractions. Each fraction was then estimated via EpiDISH algorithm, and the VIF change was calculated after adding one cell type fraction. This process was repeated for all 12 immune cell types to isolate the effects of collinearity that is driven by the shifts in leukocyte subsets. Spearman correlation was also computed to assess the relationship between each IR-mFSS-selected CpG and the estimated proportion of each immune cell type to further characterize their relationship.

**Fig. 4:**
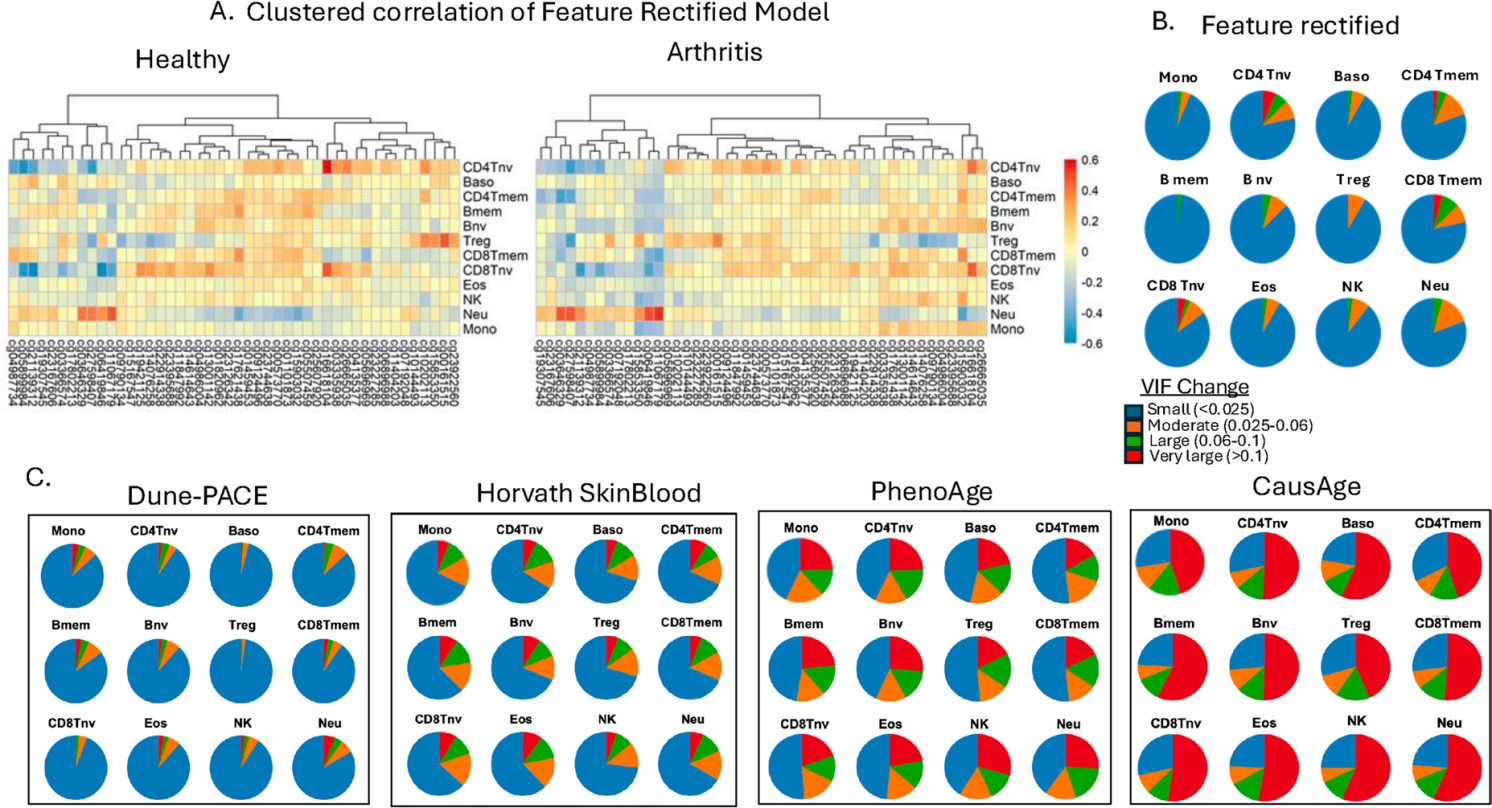
Clocks are skewed for CpGs that mark neutrophils and other subsets of leukocytes, feature rectification yields more balanced linear models with better detection of inflammaging. **A** Spearman correlation between IR-mFSS-selected CpGs and leukocyte fractions estimated via EpiDish across external arthritis dataset GSE42861 (*n* healthy = 337, *n* disease = 354). **B** Pie charts show change in VIF for IR-mFSS-selected CpGs after incorporating each leukocyte type, relative to the baseline VIF based on dataset GSE40279 of healthy individuals (*n* = 656). **C** Comparison of CpG collinearity with leukocyte fractions across different DNAm clock models, quantified by VIF changes. VIF changes were binned into categories—illustrative thresholds (“small,” “moderate,” “large,” “very large”) to visualize the distribution of CpGs with increased multicollinearity upon introducing cell-type fractions

The results demonstrate that only a small proportion of CpGs of the feature rectified model [1] correlates with different subsets of blood cells, particularly naive T cells, the decline in which is a hallmark of aging [22] (Fig. 4A, B). The majority of the IR-mFSS CpGs show minimal association (most Spearman’s rank correlations are under 0.4), and most of the IR-mFSS CpGs showed minimal VIF increases (< 0.025), suggesting they capture disease- and aging-related changes of DNAm in a comprehensive and balanced fashion, rather than purely counting the shifts in blood cell composition (Fig. 4B). In contrast, the conventional clocks, i.e., those with feature incoherence, had higher VIF increases (> 0.05 and > 0.1) in a greater proportion of the CpGs which were selected by these models, demonstrating a dependence on counting neutrophils and other leukocyte fractions (Fig. 4C).

Thus, the claims of [4] are not only incorrect but are opposite to the actual data. Interestingly, a strong association with leukocyte fractions does not allow the conventional, incoherent models to resolve the donors with age-associated diseases from healthy people [1, 2]. Hence, inflammaging might be driven by changes in gene expression of individual cells more so than by the changes in sub-set composition.

## Non-linear ML models for biological age

As compared to EN, non-linear machine learning models have a better flexibility for capturing the complexity of aging. However, they could also use biologically arbitrary data ranking, and the biomedical validity of their outputs remains untested.

AltumAge is a deep learning model, which was developed using 142 publicly available datasets on various human tissues. In contrast to EN that has ~ 200 features, AltumAge has 20,318 CpG sites to estimate biological age, and it is more accurate than linear models at older ages [28]. SHAP (SHapley Additive exPlanations) is used to deem some CpGs influential from the ML perspective. Notably, many features ranked at the top by this model reside outside of gene regulatory regions, and no mechanistic link has been established between these methylation changes and age-related phenotypes. Thus, while the model’s predictions are statistically impressive, their biological interpretability remains unknown.

XAI-AGE forces alignment of specific CpG loci with 3007 known biological processes, manually curated from the Reactome Pathway Knowledgebase [29]. Such data selection is aimed at improving the biological relevance; however, such relevance would be established only if there is evidence of an actual signaling pathway change when a given CpG beta value changes in a sample to yield a prediction of age acceleration or deceleration.

DeepAge uses temporal convolutional networks (TCNs) to model methylation as sequential data, based on the concept of epigenetic memory. However, without longitudinal validation or biological verification, the model’s assumptions remain theoretical. Furthermore, its performance is typically evaluated using an internal 80/20 data split from a single experiment, with no out-of-sample generalizability [30].

GP-age is based on Gaussian process regression, representing a probabilistic approach with a set of 30 CpGs, which was developed through DNAm analyses of 11,910 blood samples. The model produces confidence intervals alongside predictions. Nevertheless, as with all other linear and non-linear models, its chosen CpGs are selected in a biologically arbitrary way, for statistical performance, and the uncertainty estimates reflect model variance rather than biological robustness. Without mechanistic evidence, the model’s outputs, although precise, lack biomedical interpretability [31].

## Revisiting assumptions: biological aging is captured by molecular variability rather than average marker levels

From the standpoint of biomedical significance, linear predictors tend to minimize or even discard molecular features that are important for understanding the differences between healthy individuals and those with diseases and are of the same age. Linear predictors also erase the plateaus of health where aging differences are compressed or seem less visible, as well as the critical points of transition between a health plateau to a progression of biological age.

In contrast to the ML methods for biologically arbitrary feature selection and approximation, we took a different approach to uncover a natural curve of aging. Instead of attempts to equate the progression of aging with levels of biological macromolecules that are masked by natural dynamics and heterogeneity, the focus on variability itself identified it as a physiologic biomarker of aging and disease [2]. Specifically, in DNAm data, the biomarker of aging resides not within the beta values or methylation levels per se, but rather within the inherent noise captured by metrics such as coefficient of variation (CV), or deviations from the mean [2].

Even highly regulated CpGs located in genes with vitally important homeostatic functions become dysregulated with aging and disease. These CpGs maintain nearly constant mean beta values over time but have a progressive increase in standard deviations with age [2]. Importantly, our *noise barometer* identifies a natural curve of biological aging which appears to be non-linear: noise accelerates from age ~ 25 to age 36, followed by a plateau between ages 36 and 48, followed by increased aging to age 60, with various shifts occurring between ages 60 and ~ 80. Disease further alters these trajectories, increasing noise early on, which implies a relatively older biological age [2]. The notion of biological noise has been repeatedly demonstrated at the epigenetic, transcriptomic, and proteomic levels [32–34], but a noise-based discovery of the natural curve of biological aging was pioneered in Mei et al. [2].

Importantly, the noise detecting DNAm loci that identify the ages for healthy plateaus and upward shifts to risk of diseases [2] *mechanistically* underpin the phenotypic pattern of human aging that was later seen from cataloging clinical metrics [35] and has been long generally known from real-life observations.

Of note, because the magnitude of biological noise is consistently and robustly lower for the first (young) point of biological age versus old, and clock models can be applied to the stochasticity itself, using noise as yet another correlate of time progression [20] without uncovering any underlying pattern.

Quantifying biological noise as a measure of aging and disease is grounded in the mechanism of this paradigm, including not only the levels but the length of mRNA transcripts, [36]. Furthermore, there is experimental evidence of loci-specific susceptibility to biological noise, the details of which are yet to be understood [37].

Comparative proteomics also discerned the aging-related increase in variability or noise in protein levels, which additionally was increased by age-associated diseases and attenuated by a rejuvenative approach of old plasma dilution [38]. In a similar vein, studies demonstrate that inter-individual variation in specific genes is closely associated with aging progression, mirroring and supporting the observations at the epigenetic level [39]. Further supporting the tissue-specific manifestations of biological noise, variations in differentially expressed genes across different cell types and tissues of healthy individuals were shown to increase with aging [33].

Summarily, these findings suggest that the conventional comparative analyses, which focus on mean differences, may miss the key biomarker of biological aging: the increased variance, or noise, across molecules in a cell, cells in a tissue, or among individuals, and may overlook the nonlinear trajectories of aging-related changes [37–39].

Much remains to be understood with respect to biological noise, including the best methods for its quantification, particularly because it can be distinct per cell and tissue type and is influenced not only by time progression but by genetics, behavior, lifestyle, etc. environmental attributes. Therefore, although biological noise is likely a marker of biological age, further systematic studies are needed to uncover its underlying mechanisms and standardize its measurements.

In summary, most current ML models of aging are statistically effective in approximating time progression and are not accurate for determinations of biological age due to the erroneous assumption that the natural patterns can be transformed without a biological justification. The biomedical interpretability of age clocks is lacking, particularly because it is most important to understand the non-linear and increasingly variable with age epigenetics, gene expression, proteins, and metabolites. Variations that are due to genetics, natural dynamics, habits, and environments dominate the distinct, incremental changes in the levels of most/all studied biomolecules across age groups. Even genes and proteins with stable mean values over time (homeostatically vital) could have increasingly high variations with age, reflecting a loss of regulatory stability and increased biological entropy. Biological noise is emerging as the true, and possibly, the only quantifiable biomarker of biological aging.

## Data Availability

Data used for multi-collinearity and coherence analysis are GSE40279 and GSE42861. The same datasets of rheumatoid arthritis as in [1, 2] were used to comparatively profile the skewing of the models toward leukocyte subsets, directly testing the claims and assumptions of [4].

## Code availability

Code used to generate Fig. 4, as well as the relevant figures for the website, is published in the GitHub Repository github.com/SerafinaMei/Misalignment_of_Age_Clocks. Cell-type fractions in epigenome data were estimated and corrected using EpiDISH.
